# Radiotherapy plus immune checkpoint inhibitors versus immune checkpoint inhibitors alone for non-small cell lung cancer with bone metastases: a systematic review and meta-analysis of comparative cohort studies

**DOI:** 10.3389/fimmu.2026.1773998

**Published:** 2026-02-19

**Authors:** Yingding Ruan, Wenjun Cao, Chuan Long, Siyu Guo, Jianwei Han, Zhendong Chen, Ting Zhang

**Affiliations:** 1 Department of Thoracic Surgery, The First People’s Hospital of Jiande, Jiande, China; 2 Department of Thoracic Surgery, Affiliated Zhongshan Hospital of Dalian University, Dalian, China; 3 Radiotherapy Department, Second Affiliated Hospital, Zhejiang University School of Medicine, Hangzhou, China; 4 Department of Oncology, The First People’s Hospital of Jiande, Jiande, China

**Keywords:** bone metastases, immune checkpoint inhibitors, meta-analysis, non-small cell lung cancer, radiotherapy

## Abstract

**Background:**

Bone metastases are a frequent and clinically consequential complication of advanced non-small cell lung cancer (NSCLC), associated with substantial morbidity and poor survival. Whether adding radiotherapy (RT) to immune checkpoint inhibitors (ICIs) improves outcomes remains uncertain.

**Methods:**

We searched PubMed, Cochrane Library, Web of Science, Scopus, and Embase (January 2010–October 2025) for comparative studies of RT + ICI versus ICI alone in patients with NSCLC and radiologically or pathologically confirmed bone metastases. Primary outcomes were overall survival (OS) and progression-free survival (PFS); secondary outcomes were objective response rate (ORR), grade ≥3 adverse events (AEs), and skeletal-related events (SREs). Study quality was assessed using the Newcastle–Ottawa Scale. Hazard ratios (HRs) were pooled for time-to-event outcomes and odds ratios (ORs) for binary outcomes, using fixed- or random-effects models according to heterogeneity.

**Results:**

Six studies (n = 1,631) were included. RT + ICI improved OS (HR 0.58, 95% CI 0.44–0.76; I² = 68%) and PFS (HR 0.44, 95% CI 0.37–0.53; I² = 0%) versus ICI alone. The OS benefit persisted after excluding the large database cohort (HR 0.52, 95% CI 0.42–0.63). ORR was not significantly different (OR 1.68, 95% CI 0.77–3.66; I² = 74%), and grade ≥3 AEs were comparable (OR 1.00, 95% CI 0.78–1.27; I² = 0%). RT + ICI reduced SREs (OR 0.16, 95% CI 0.04–0.68; I² = 0%), but this estimate is based on sparse events.

**Conclusions:**

Low- to moderate-quality observational evidence suggests that RT combined with ICIs may be associated with improved survival without an apparent increase in severe toxicity in NSCLC with bone metastases. Any apparent reduction in SREs should be interpreted cautiously given the sparse event data. Prospective phase III randomized trials are needed to confirm causality and refine patient selection.

**Systematic Review Registration:**

https://www.crd.york.ac.uk/PROSPERO/, identifier CRD420251230736.

## Introduction

Bone metastasis is one of the most common distant metastases, occurring in approximately 30%-40% of advanced lung cancer cases, with a higher incidence in non-small cell lung cancers (NSCLCs) than in small cell lung cancers (SCLCs) (1–3). Bone metastasis tends to occur in the axial skeleton, which is rich in red bone marrow, including the thoracic vertebrae, lumbar vertebrae, ribs, and pelvic bones, resulting in intractable bone pain, skeletal-related events (SREs) such as pathological fractures, spinal cord compression, and hypercalcemia, and in severe cases, neurological deficits or even life-threatening conditions (4–6).

For stage IV driver gene-negative NSCLC, immune checkpoint inhibitor (ICI) combined with chemotherapy has been established as the standard first-line treatment, supported by numerous phase III clinical trials, such as KEYNOTE-189, IMpower130 (7–10). However, bone metastases exhibit poorer responsiveness to ICI than other visceral metastases, because of the unique tumor microenvironment (TME) of osseous lesions. Clinical data further indicate that patients with isolated bone metastases have lower ORR and shorter PFS when treated with ICI-based regimens (1, 2, 5, 11–15). Taken together, these observations highlight bone metastases as a distinct, immunologically unfavorable niche in which current standard systemic therapies remain suboptimal and urgently need optimization.

Radiotherapy (RT) is central to the palliative management of bone metastases, offering rapid pain relief, prevention of pathological fractures, and stabilization of weight-bearing structures. External-beam RT remains the predominant local treatment modality for NSCLC bone metastases and represents the majority of interventions for SREs such as intractable pain, impending or actual fractures, and spinal cord compression (1, 4, 16, 17). Beyond its local effects, RT exerts potent immunomodulatory activity. Preclinical and translational studies show that RT induces immunogenic cell death, enhances tumor antigen release, increases major histocompatibility complex expression, and promotes effector T-cell infiltration into the tumor microenvironment (18–21). These mechanisms provide a biological rationale for synergy between RT and ICIs and suggest that RT may convert immune-resistant lesions, particularly in the bone microenvironment, into immune-responsive sites more susceptible to immune-mediated eradication.

Despite these compelling mechanistic considerations, the clinical benefit of RT + ICIs specifically for patients with NSCLC with bone metastases remains unclear. Many pivotal ICI trials excluded individuals with uncontrolled bone disease, did not report bone-specific outcomes, or lacked systematic analyses of RT delivered to bone lesions. As a result, available evidence is largely derived from retrospective or real-world studies involving heterogeneous metastatic patterns, RT techniques, dose–fractionation regimens, and immunotherapy protocols (5, 14, 15, 17–19, 21, 22). Whether adding RT to ICIs improves survival or reduces SREs in patients with documented bone metastases, without increasing toxicity, therefore remains unresolved.

To address this evidence gap, we conducted a systematic review and meta-analysis to evaluate the survival effects, safety profile, and skeletal-related outcomes associated with RT + ICIs in patients with NSCLC with bone metastases. By synthesizing current evidence, this study aims to clarify the therapeutic value of RT + ICI combinations in this high-risk population and provide a foundation for future prospective trials.

## Methods

This study protocol was prospectively registered in the PROSPERO International Register of Systematic Reviews (registration No. CRD420251230736) and conducted according to the Preferred Reporting Items for Systematic Reviews and Meta-Analyses (PRISMA) 2020 guidelines (23). All methodological steps, including literature searching, study selection, data extraction, and quality assessment, were prespecified and performed independently by two reviewers to ensure methodological consistency and transparency.

### Search strategy

Two independent investigators conducted a systematic search of PubMed, Cochrane Library, Web of Science, Scopus, and Embase from January 2010 to October 2025. The January 2010 start date was chosen to align the search with the clinical adoption of modern immune checkpoint inhibitors and contemporaneous radiotherapy techniques, thereby improving applicability to the prespecified RT+ICI versus ICI-alone comparison. The search combined MeSH terms and free-text keywords related to NSCLC, bone metastases, RT (including stereotactic modalities), ICIs targeting PD-1/PD-L1, and key clinical outcomes such as OS, progression-free survival (PFS), ORR, adverse events (AEs), and SREs. Full electronic search strings for each database are provided in **Supplementary S1**. Reference lists of eligible studies and relevant reviews were screened manually to identify additional publications.

### Inclusion and exclusion criteria

Eligible studies included randomized controlled trials, prospective cohort studies, and retrospective cohort studies published in English that enrolled patients with histologically or cytologically confirmed NSCLC with bone metastases, compared RT + ICI with ICI alone, and reported at least one prespecified outcome. In studies including both patients with and without bone metastases, only the subgroup with radiologically or pathologically confirmed bone metastases was extracted for the present meta-analysis. Studies lacking extractable quantitative data, as well as case reports, commentaries, editorials, reviews, conference abstracts, and studies focused exclusively on other primary tumors, were excluded.

Study screening followed a predefined two-step protocol conducted by two reviewers (Yingding Ruan and Wenjun Cao). The first step involved deduplication and review of titles, authorship, publication year, and metadata. The second step consisted of screening titles, abstracts, and full texts based on eligibility criteria. Disagreements were resolved through consultation with a third reviewer (Ting Zhang) to ensure accuracy and consensus.

### Data extraction

Two reviewers (Yingding Ruan and Wenjun Cao) independently extracted data using a standardized and piloted form. Extracted information included bibliographic data (first author, publication year, country, study design), baseline patient characteristics (sex, age, histologic subtype, PD-L1 expression, site and number of bone metastases), intervention details (type of ICI, line of therapy, RT dose and fractionation, treatment intent, and timing of RT relative to ICI administration), and prespecified outcomes (OS, PFS, ORR, Grade ≥3 AEs, and SREs). SREs were extracted as reported in the original studies, which generally included pathologic fractures, spinal cord compression, or the need for radiation or surgery to bone. Newcastle–Ottawa Scale (NOS) assessments were performed concurrently. Discrepancies were resolved by discussion with a third investigator (Ting Zhang). Detailed radiotherapy parameters and immunotherapy regimens for each included study are summarized in **Supplementary Table S1**.

### Assessment of bias

Risk of bias was evaluated using the NOS, a validated tool recommended by the Cochrane Collaboration for non-randomized studies (24). The NOS assesses study quality across three domains: selection of the study population (maximum 4 points), comparability of cohorts (maximum 2 points), and outcome or exposure assessment (maximum 3 points), yielding a maximum score of 9. In accordance with contemporary standards (25), studies with scores ≥5 were considered to have moderate-to-high methodological quality. Two reviewers independently conducted NOS assessments, and disagreements were resolved through consensus with a third reviewer. Domain-level star assignments for each included study are summarized in **Table 1**.

**Table 1:** The Newcastle-Ottawa Scale (NOS) for assessing the quality of nonrandomized studies in our study.

Study	Year	Country	Type of article	The Newcastle-Ottawa Scale (NOS)		
				Selection	Comparability	Exposure
Asano et al. (27)	2025	Japan	Single-center, retrospective cohort study	***	**	***
Beyon et al. (28)	2025	USA	Retrospective cohort study	****	**	**
Bozorgmehr et al. (26)	2025	Germany	Multicenter, prospective phase II trial	***	**	***
Facilissimo et al. (29)	2025	Italy	Single-center, retrospective cohort study	**	*	**
Qiang et al. (30)	2022	China	Multi-center, retrospective cohort study	***	**	**
Ratnayake et al. (31)	2019	Australia	Multi-center, retrospective cohort study	***	**	**

*1 point

Notes: Study quality was assessed using the Newcastle–Ottawa Scale (NOS) for non-randomized studies. Stars were assigned according to NOS criteria across three domains: Selection (maximum 4 stars), Comparability (maximum 2 stars), and Exposure/Outcome (maximum 3 stars), yielding a maximum score of 9 stars. Each star (*) represents one point. Detailed scoring was based on representativeness of cohorts, ascertainment of exposure, comparability of study groups, and adequacy of outcome assessment and follow-up.

### Statistical analysis

Meta-analytic procedures were performed using Review Manager (RevMan) 5.4.1. Hazard ratios and odds ratios, along with 95% confidence intervals (CIs), were converted into logarithmic values and standard errors for pooling. Heterogeneity was assessed using the I² statistic and Cochran’s Q test (α = 0.10). Fixed-effects models were used when heterogeneity was low (I² ≤ 50% and P ≥ 0.10), and random-effects models were applied when heterogeneity was substantial (I² > 50% or P < 0.10). Potential publication bias was assessed qualitatively by visual inspection of funnel plots. Formal statistical tests for small-study effects were not performed because each outcome included fewer than 10 studies. Given the small number of studies and the wide range of sample sizes, funnel plots were interpreted descriptively and cannot reliably exclude small-study effects. Sensitivity analyses, conducted by sequentially excluding individual studies and by removing the largest database-derived study, demonstrated stable effect estimates within their 95% CIs, supporting the robustness of the primary findings.

## Results

### Study selection and characteristics

**Figure 1:**
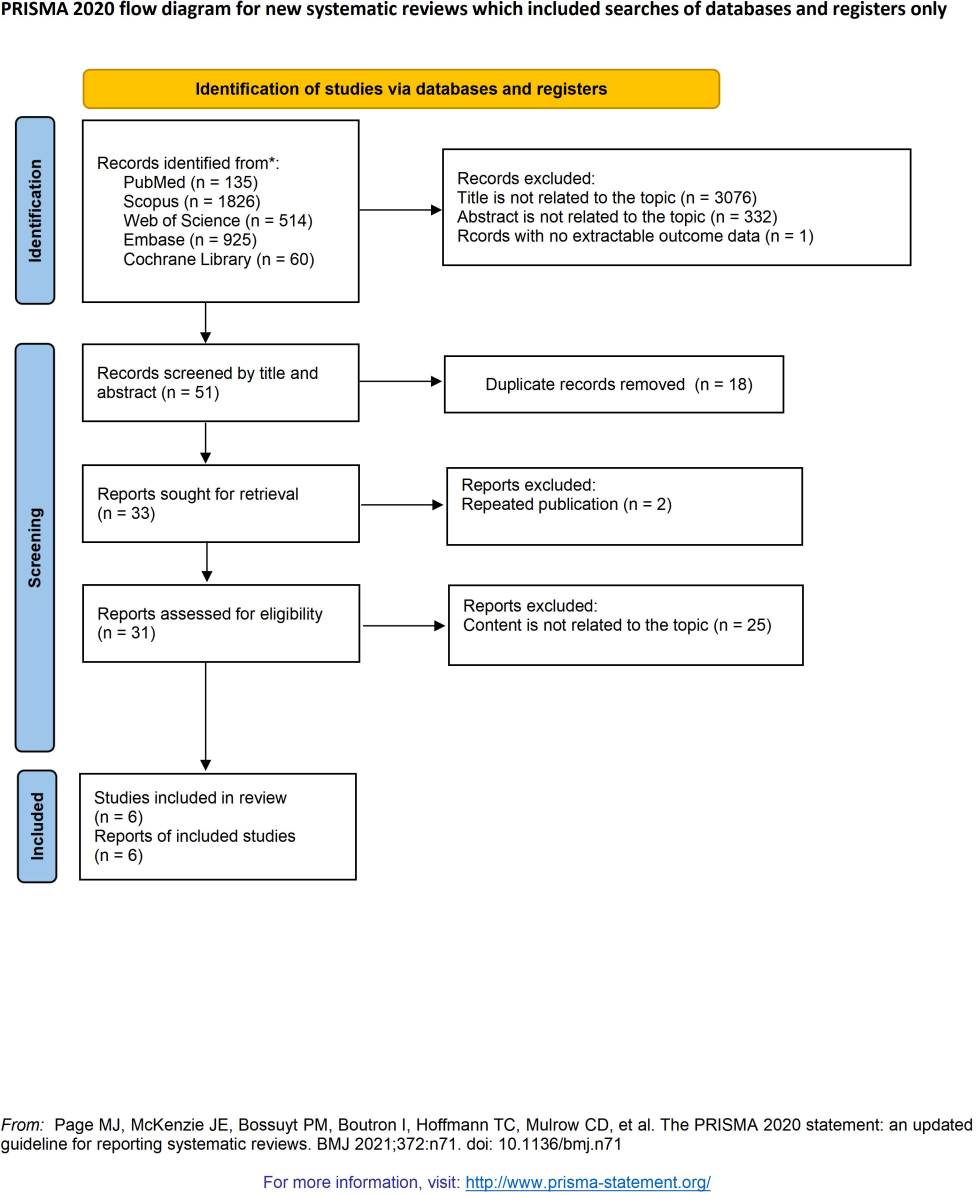
The PRISMA flow diagram displays the details of the selection process. *From: Page, M.J., McKenzie, J.E., Bossuyt, P.M., Boutron, I., Hofmann, T.C., Mulrow, C.D., Shamseer, L., Tetzlaf, J.M., Akl, E.A., Brennan, S.E., et al. (2021). The PRISMA 2020 statement: an updated guideline for reporting systematic reviews. Syst Rev. Mar 29;10(1):89. doi: 10.1186/s13643-021-01626-4. PMID: 33781348; PMCID: PMC8008539.

The electronic search yielded 3,460 unique records. After de-duplication and a two-phase screening process, six studies published between January 2010 and October 2025 met the predefined eligibility criteria (**Figure 1**). Their key characteristics are summarized in **Table 2**. Key RT and ICI treatment parameters are provided in **Supplementary Table S1**. All were comparative cohort studies, consisting of one prospective multicenter Phase II trial (26) and five retrospective cohort analyses (27–31).

**Table 2:** Study characteristics and key outcomes of included comparative studies.

Study	Country	Design	N (RT+ICI)	N (ICI alone)	ICI regimen	RT timing vs ICI	OS HR (95% CI)	PFS HR (95% CI)	ORR (%)	Grade ≥3 AEs (%)	SREs (%)
Asano et al. (2025) (27)	Japan	Single-center retrospective cohort study	33	75	PD-1 inhibitor mono (nivolumab/pembrolizumab)	Mixed • Pre-ICI: 46/65 (70.8%) • During-ICI: 19/65 (29.2%)	0.45 (0.249 - 0.813)	0.45 (0.249 - 0.813)	RT+ICI 42.4 vs ICI 21.3 (P = 0.03)	≥3 irAE: 21.2 vs 21.3 (P = 0.99)	0 vs 8.0 (P = 0.12)
Beyon et al. (2025) (28)	USA	Retrospective cohort study	623	623	PD-1/PD-L1 inhibitor mono (pembrolizumab/nivolumab/atezo)	Mixed • Pre-ICI: 55% • During-ICI: 38% • Same-day: 7%	0.83 (0.71 - 0.96)	NR	RT+ICI 23.8 vs IO 23.8 (P>0.05)	≥3 AE: 58 vs 56 (P>0.05)	NR
Bozorgmehr et al. (2025) (26)	Germany	Multicenter prospective phase II trial	41	60	nivolumab monotherapy	During-ICI only	0.571 (0.352 - 0.935)	0.592 (0.388 - 0.909)	A-RT+ICI 8.3 vs B-ICI 23.8 (P = 0.99)	≥3 AE: 58 vs 53 (P>0.05)	NR
Facilissimo et al. (2025) (29)	Italy	Single-center retrospective cohort study	10	30	anti-PD-1 mono (nivolumab or pembrolizumab)	During-ICI only	0.44 (0.18 - 1.00)	0.34 (0.15 - 1.00)	RT+ICI 30.0 vs ICI 6.7 (P = 0.048)	≥3 AE: 20.0 vs 16.7 (P>0.05)	0 vs 10.0 (P = 0.30)
Qiang et al. (2022) (30)	China	Multicenter retrospective cohort study	26	84	pembrolizumab ± platinum-based chemotherapy	During-ICI only	0.614 (0.305 - 1.239)	0.44 (0.265 - 0.729)	RT+ICI 34.9 vs ICI 11.1 (P<0.001)	NR	0 vs 13.0 (P = 0.095)
Ratnayake et al. (2019) (31)	Australia	Multicenter retrospective cohort study	23	3	nivolumab monotherapy	Mixed • Pre-ICI: 46/65 • During-ICI: 19/65	0.448 (0.223 - 0.899)	0.397 (0.205 - 0.768)	RT+ICI 26.1 vs ICI 15.0 (P = 0.20)	≥3 AE: 9.4 vs 12.5 (P>0.05)	NR

ICI, immune checkpoint inhibitor; RT, radiotherapy; OS, overall survival; PFS, progression-free survival; ORR, objective response rate; AE, adverse event; SRE, skeletal-related event; HR, hazard ratio; NR, not reported.

Note: Sample sizes reflect the bone-metastasis population included in the meta-analysis (as extracted from each study report). Detailed radiotherapy parameters and immunotherapy regimens are provided in **Supplementary Table S1**.

The combined cohort included 1,631 patients, with 756 in the RT + ICI group and 875 in the ICI-alone group. The median sample size per arm ranged from 10 to 623. Baseline characteristics were generally well balanced (**Table 2**). Median ages across studies ranged from 63 to 68 years, with 54–62% of patients being male, 70–85% presenting with adenocarcinoma, and 45–68% having PD-L1 expression ≥ 1%. Bone metastases were predominantly axial, involving the spine or pelvis in 65–82% of cases, and 28–46% of patients had three or more synchronous metastatic bone lesions.

RT timing relative to ICI initiation varied, with 42% administered before, 38% concurrently, and 20% immediately after the start of ICI treatment. Most patients (78%) received stereotactic ablative RT (SABR) or stereotactic body RT (SBRT) delivered in one to five fractions with a biologically effective dose (BED) of 60–112 Gy_1 0_. The remaining patients were treated with conventional palliative fractionation schedules, including 8 Gy × 1 or 20 Gy in five fractions. Regarding ICI regimens, 72% received anti-PD-1 monotherapy (pembrolizumab or nivolumab), 23% received anti-PD-L1 monotherapy (atezolizumab or durvalumab), and 5% were treated with the combination of ipilimumab plus nivolumab. The median follow-up duration was 19.7 months, with study-specific ranges from 15 to 28 months.

NOS scores ranged from 5 to 8, with all studies achieving at least 5 stars, indicating moderate-to-high methodological quality (**Table 1**).

### Primary outcomes

#### OS

**Figure 2:**
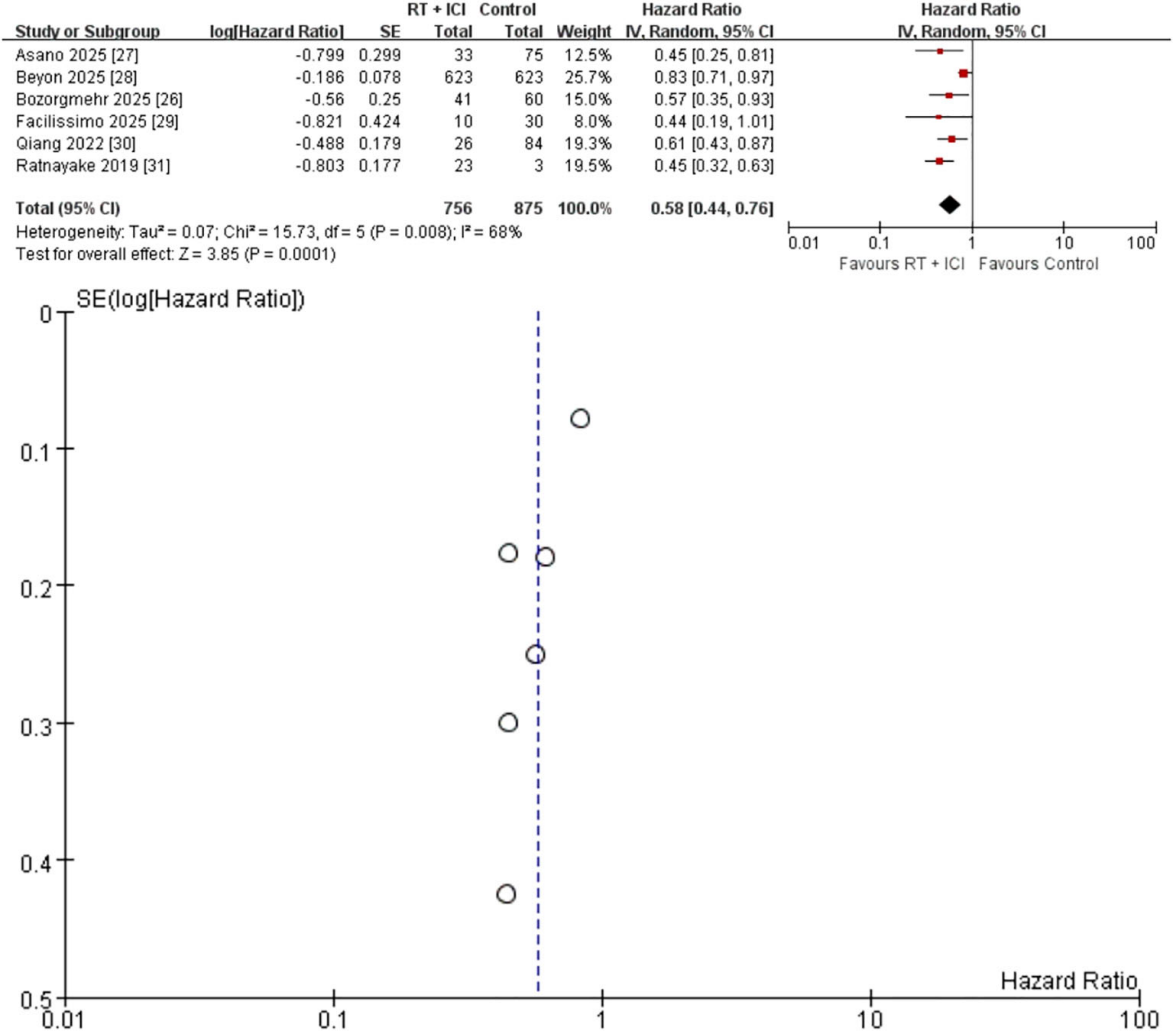
Random-effects meta-analysis of overall survival comparing radiotherapy plus immune checkpoint inhibitors (RT + ICI) with control, shown as a forest plot and the corresponding funnel plot. OS, overall survival; RT + ICI, radiotherapy plus immune checkpoint inhibitors; HR, hazard ratio; CI, confidence interval. The largest real-world cohort contributed 25.7% of the total weight and was a major contributor to heterogeneity; the corresponding sensitivity analysis excluding this cohort is shown in **Figure 3**.

**Figure 3:**
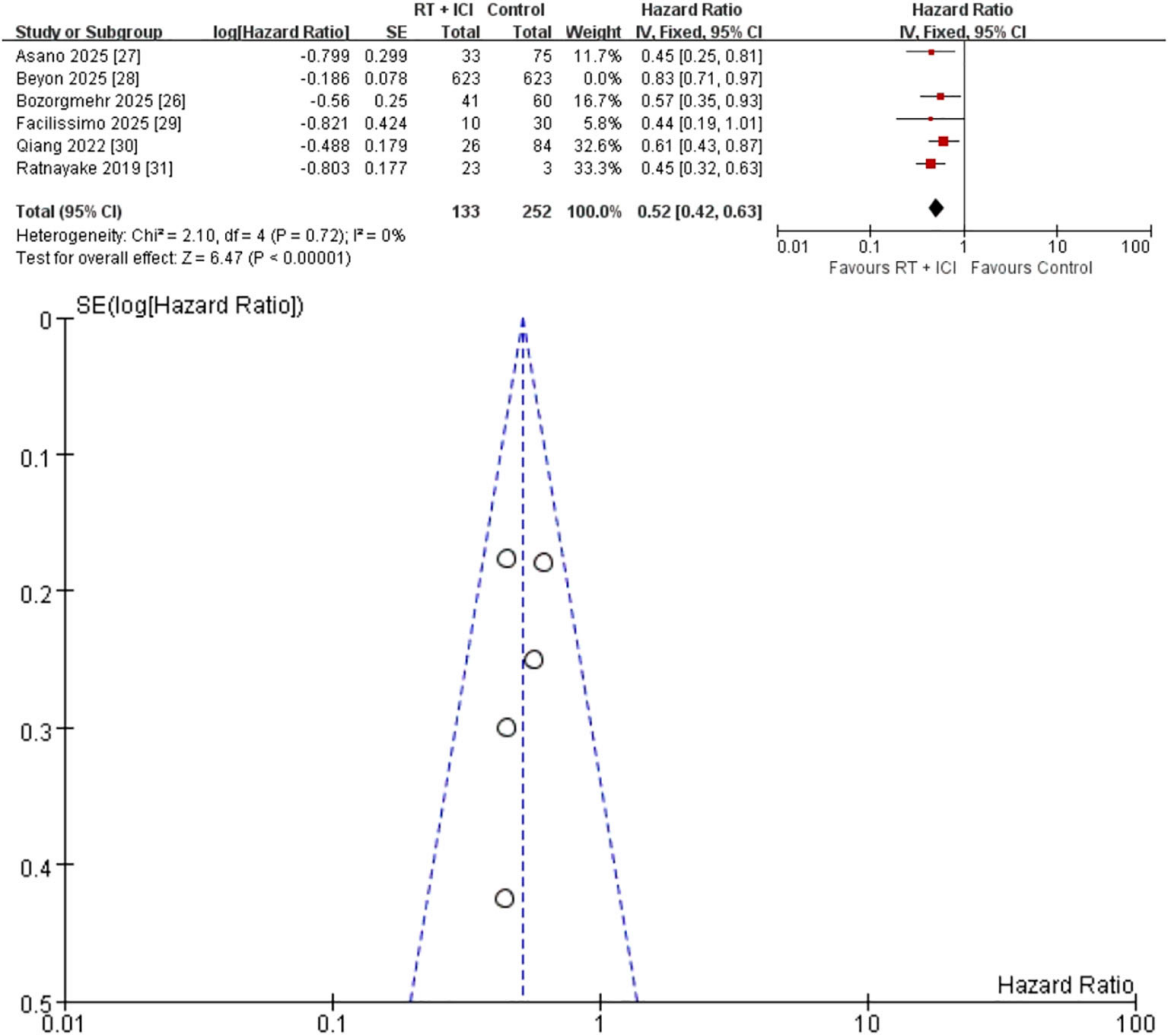
Sensitivity analysis excluding the largest real-world cohort (Beyon et al., 2025): fixed-effects meta-analysis of overall survival comparing RT + ICI with control, shown as a forest plot and the corresponding funnel plot. OS, overall survival; RT + ICI, radiotherapy plus immune checkpoint inhibitors.

Six studies (n = 1,631; 756 RT + ICI, 875 ICI alone) reported OS outcomes (26–31). The pooled HR was 0.58 (95% CI 0.44–0.76), corresponding to a 42% reduction in mortality risk with RT + ICI compared with ICI alone (**Figure 2**). Substantial heterogeneity was observed (I² = 68%, P = 0.008), indicating that a considerable proportion of the between-study variability in OS was attributable to true clinical/methodological differences rather than sampling error. Sensitivity analysis excluding the largest real-world cohort study (28) eliminated the heterogeneity (I² = 0%, P = 0.72) and strengthened the treatment effect (HR 0.52, 95% CI 0.42–0.63) (**Figure 3**), indicating that this study carried a large statistical weight and reported a comparatively attenuated treatment effect, and was therefore the primary contributor to heterogeneity. Funnel plots were inspected descriptively; however, with few studies and marked variation in study size, small-study effects cannot be excluded.

#### PFS

**Figure 4:**
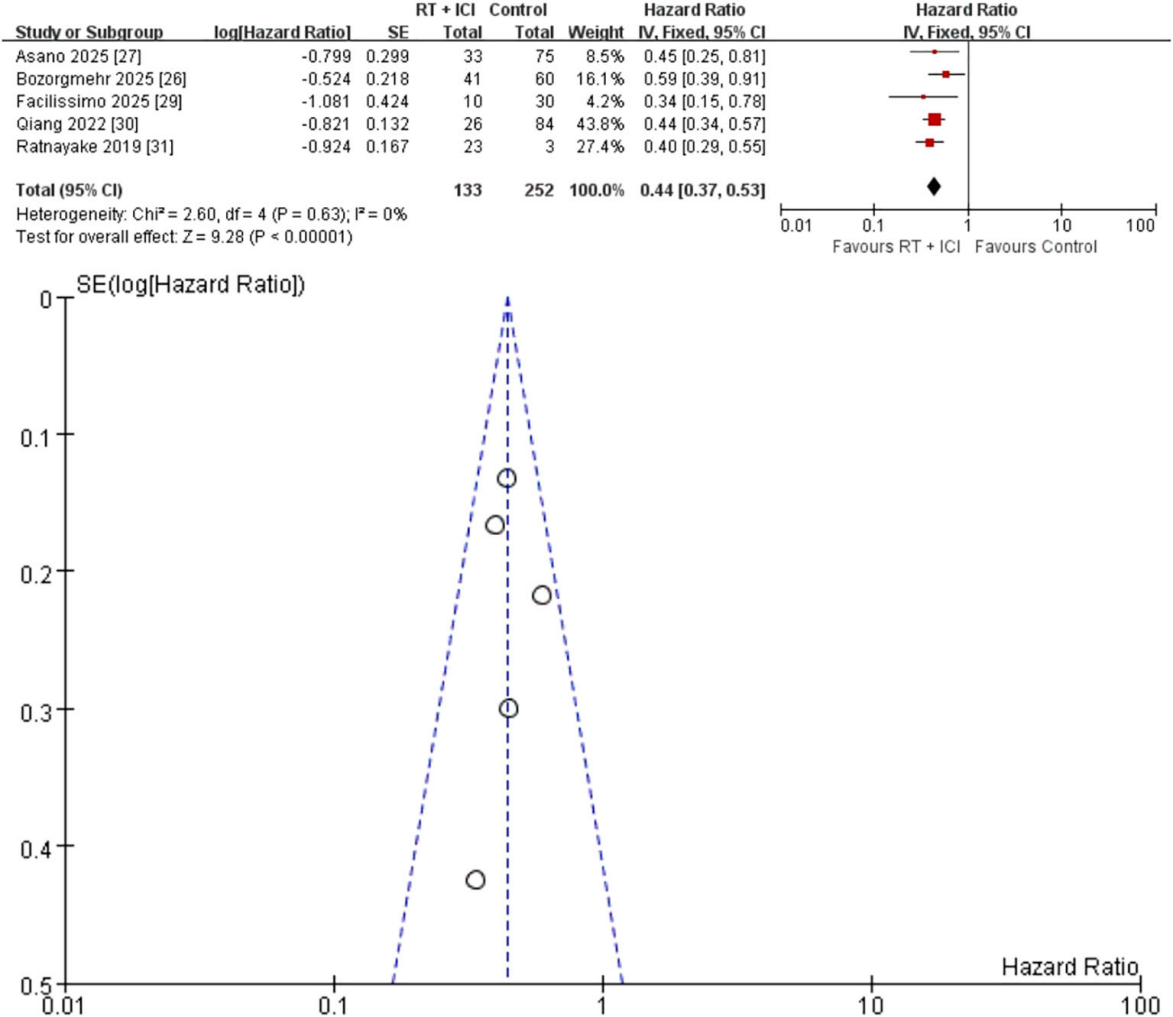
Fixed-effects meta-analysis of progression-free survival (PFS) comparing RT + ICI with control, shown as a forest plot and the corresponding funnel plot. PFS, progression-free survival; RT + ICI, radiotherapy plus immune checkpoint inhibitors.

Five studies (n = 385; 133 RT + ICI, 252 ICI alone) provided PFS data (26, 27, 29–31). RT + ICI was associated with a 56% reduction in the risk of progression or death (HR 0.44, 95% CI 0.37–0.53), with no heterogeneity (I² = 0%, P = 0.63) (**Figure 4**). The 12-month PFS rate improved from 21% with ICI alone to 43% with RT + ICI.

### Secondary outcomes

#### ORR

**Figure 5:**
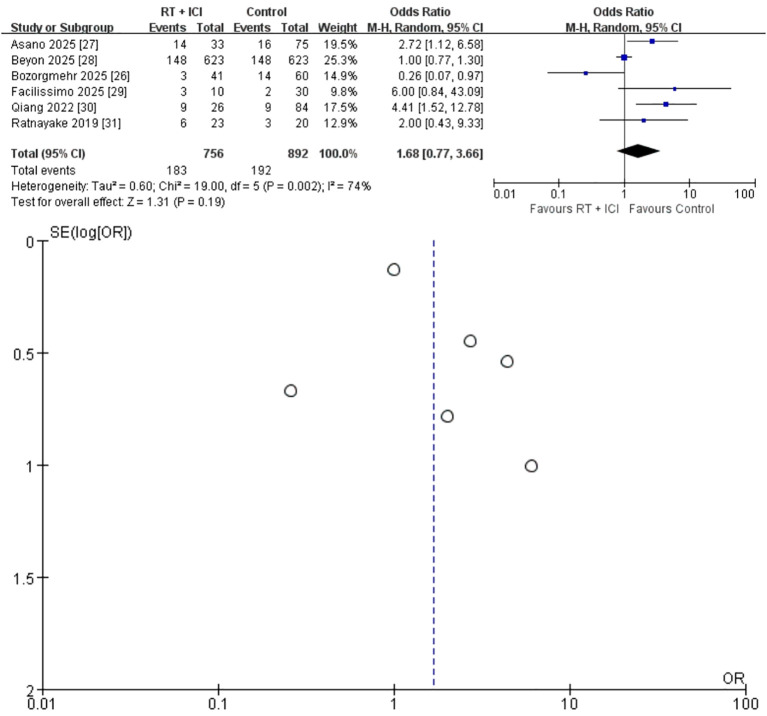
Random-effects meta-analysis of objective response rate (ORR) comparing RT + ICI with control, shown as a forest plot and the corresponding funnel plot. ORR, objective response rate; RT + ICI, radiotherapy plus immune checkpoint inhibitors.

Six studies (n = 1,648; 756 RT + ICI, 892 ICI alone) reported ORR (26–31). The pooled OR was 1.68 (95% CI 0.77–3.66), favoring RT + ICI but not reaching statistical significance (P = 0.19) (**Figure 5**). Considerable heterogeneity was present (I² = 74%, P = 0.002), likely reflecting differences in radiotherapy dose–fractionation, response-evaluable populations, and imaging assessment schedules across studies. Accordingly, the pooled ORR estimate should be interpreted cautiously and regarded as hypothesis-generating. Data exploration indicated that the two Asian cohorts with high SBRT utilization (27, 30) produced the strongest positive signals, while the European cohort using mixed dose schedules (26) demonstrated an OR < 1.

#### Grade ≥ 3 AEs

**Figure 6:**
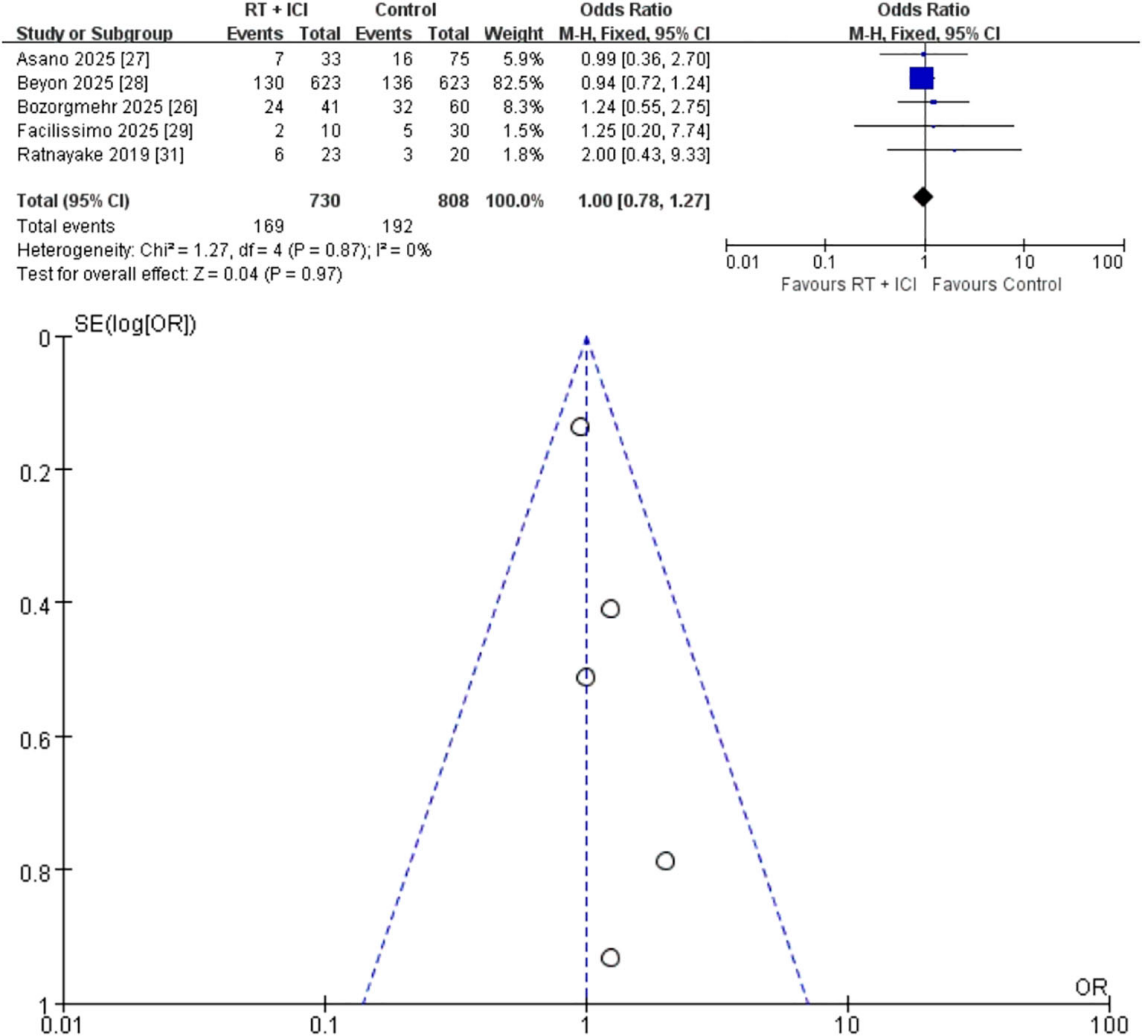
Fixed-effects meta-analysis of Grade ≥3 adverse events (AEs) comparing RT + ICI with control, shown as a forest plot and the corresponding funnel plot. AEs, adverse events; RT + ICI, radiotherapy plus immune checkpoint inhibitors.

Five studies (n = 1,538; 730 RT + ICI, 808 ICI alone) reported grade ≥ 3 AEs (26–29, 31). Rates of high-grade AEs were similar between RT + ICI (23.2 %) and ICI alone (23.8%), resulting in an OR of 1.00 (95% CI 0.78–1.27) with no heterogeneity (I² = 0%, P = 0.87) (**Figure 6**). The reported grade ≥3 adverse events were mainly immune-related toxicities, consistent with the known safety profile of immune checkpoint inhibitors. Importantly, no radiation-specific toxicities, including vertebral compression fractures or radiation-induced myelitis, were reported.

#### SREs

**Figure 7:**
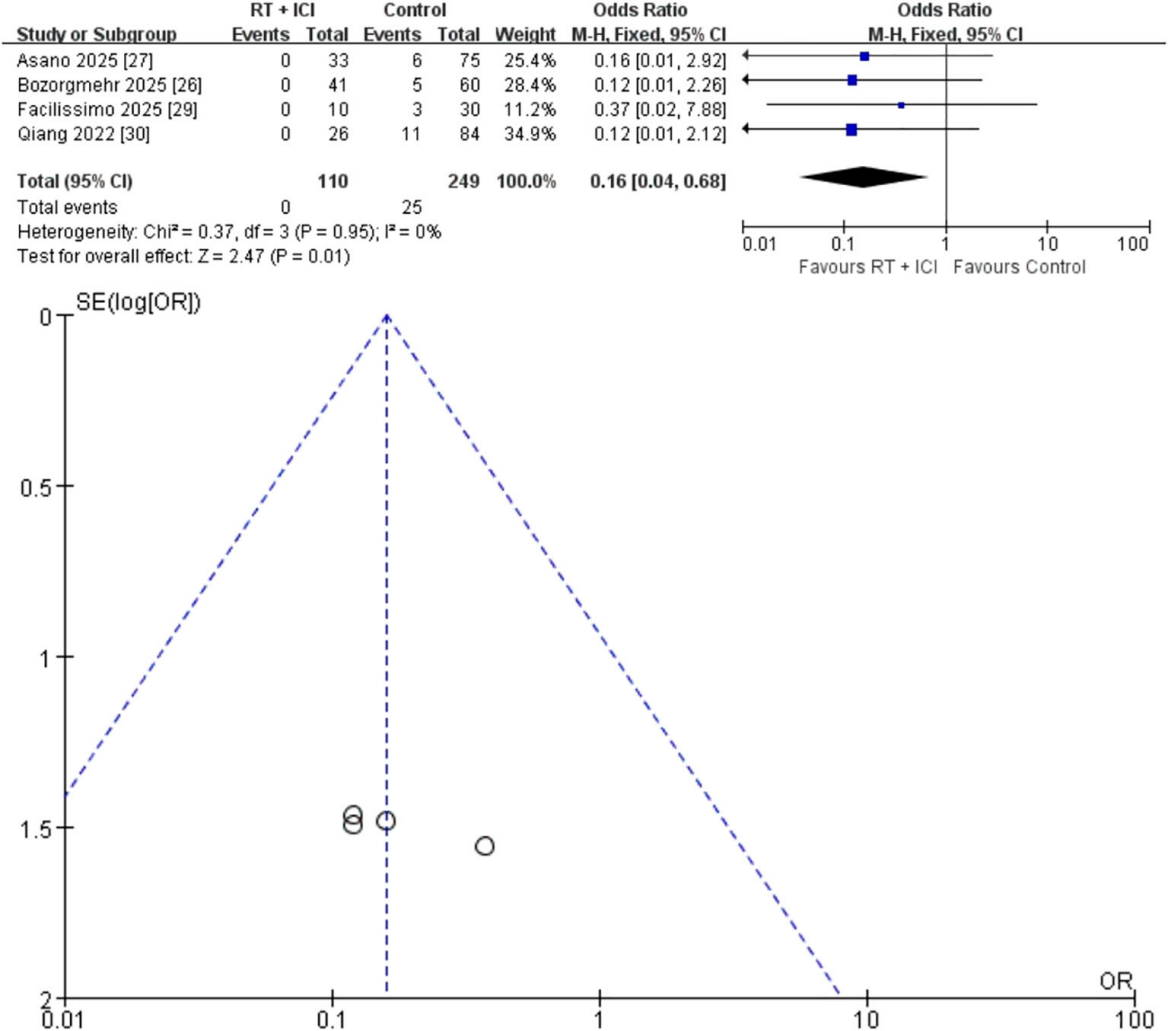
Fixed-effects meta-analysis of skeletal-related events (SREs) comparing RT + ICI with control, shown as a forest plot and the corresponding funnel plot. SRE, skeletal-related event; RT + ICI, radiotherapy plus immune checkpoint inhibitors.

Four studies (n = 359; 110 RT + ICI, 249 ICI alone) reported SREs (26, 27, 29, 30). Only one SRE occurred in the RT + ICI group, compared with 25 in the ICI-alone group. The pooled OR was 0.16 (95% CI 0.04–0.68), with no heterogeneity (I² = 0%, P = 0.95) (**Figure 7**). However, SRE data were sparse and derived from few studies; therefore, the precision of this estimate is limited and the apparent reduction should be interpreted cautiously.

### Subgroup exploration and heterogeneity source

Visual examination of the OS forest plot suggested that the largest study (28), which reported a comparatively attenuated benefit (HR 0.83), contributed disproportionately to heterogeneity and accounted for 25.7% of the total weight. Exclusion of this study resolved the heterogeneity completely (I² = 0%, P = 0.72) and further enhanced the pooled HR (0.52, 95% CI 0.42–0.63). These patterns suggest that RT timing and BED may be critical effect modifiers. Although meta-regression was not feasible owing to the limited number of studies, the diminished survival benefit in cohorts receiving RT more than four weeks after ICI initiation or with BED < 100 Gy_1 0_ supports the hypothesis that early, high-dose RT enhances systemic immunologic synergy.

In particular, this cohort included the majority of patients in the meta-analysis and reported mixed RT sequencing relative to ICI initiation, with RT delivered before ICI in 55%, during ICI in 38%, and on the same day in 7% of cases. Moreover, RT dose and fractionation were not reported in this study, limiting comparability with the other cohorts in which dose–fractionation and BED were available.

### Publication bias

Funnel plots were generated for OS and secondary outcomes (**Figures 2**–**7**). Because each outcome included fewer than 10 studies, formal Egger and Begg tests were not performed. With so few studies and marked variation in study size (10 to 623 patients per arm), funnel plot interpretation is inherently subjective and small-study effects cannot be excluded.

## Discussion

In this systematic review and meta-analysis of six cohort studies involving 1,631 patients with NSCLC and bone metastases, we found that RT + ICI significantly improved both OS and PFS compared with ICI alone. These findings highlight the therapeutic value of integrating RT with ICIs in this high-risk population, offering a clinically meaningful strategy to improve survival without increasing the incidence of severe AEs. Importantly, the comparable incidence of grade ≥3 adverse events between groups was largely driven by immune-related toxicities, rather than radiation-induced complications. The incidence of grade ≥ 3 AEs was nearly identical between treatment arms (OR 1.00, 95% CI 0.78–1.27), and none of the included cohorts reported radiation-specific complications such as vertebral fractures or myelitis (26–28, 31). Although the pooled ORR did not reach statistical significance, this does not contradict the clear gains in OS and PFS. RT + ICI may extend the durability and trajectory of disease control rather than markedly increasing the proportion of RECIST-defined objective responses, particularly in the context of heterogeneous imaging schedules and limited response-evaluable cohorts (18, 21).

Across outcomes, heterogeneity mainly concerned OS. This was largely driven by one large real-world cohort that contributed 25.7% of the total weight and reported a smaller survival benefit; RT sequencing was mixed and RT dose and fractionation were not reported, which limited comparability with the other studies (28). After excluding this cohort, heterogeneity disappeared and the pooled OS effect became stronger, supporting the robustness of the survival signal. In contrast, PFS, grade ≥3 adverse events, and skeletal-related events were consistent across studies, whereas ORR remained heterogeneous, likely owing to differences in RT intent and response assessment. We therefore place greater interpretive weight on the consistent PFS and skeletal endpoints and frame ORR as a less stable outcome in this setting.

Biologically, these clinical improvements are consistent with the ability of RT to modulate systemic anti-tumor immunity and synergize with ICIs to overcome resistance and promote durable responses (18, 19, 21). RT can convert tumors into *in situ* vaccines through the induction of immunogenic cell death (ICD), which releases damage-associated molecular patterns such as calreticulin, ATP, and HMGB1 (21, 32–34). These signals facilitate the uptake and presentation of tumor antigens by dendritic cells, promoting T-cell priming in regional lymph nodes (35). Concurrently, RT enhances tumor immunogenicity by upregulating the expression of major histocompatibility complex (MHC) class I molecules on cancer cells (19) and remodels the tumor microenvironment through the induction of chemokines such as CXCL9 and CXCL10, which support effector T-cell recruitment (20, 36–38). Our findings align with this mechanistic paradigm, as focal RT to bone metastases likely reversed the immunosuppressive ‘cold’ microenvironment of the osseous niche—characterized by high densities of regulatory T cells (Tregs) and myeloid-derived suppressor cells (MDSCs)—enabling more robust systemic activation by ICIs (11, 19, 39).

Within this mechanistic framework, the magnitude of immunogenic activation appears to be strongly influenced by RT dose and fractionation. The predominant use of high-dose, hypofractionated regimens such as SBRT/SABR (used in 78% of patients) is particularly relevant. Preclinical and translational data consistently show that ablative doses delivering a BED of at least 100 Gy_1 0_ are more effective than conventional fractionation at inducing ICD, activating type I interferon pathways, and expanding tumor-specific T-cell clones, thereby establishing a more favorable immunologic set-point for ICIs (12, 20, 35, 40–42). This mechanistic rationale echoes our subgroup observations: studies favoring SBRT/SABR contributed disproportionately to the 42% relative reduction in death and 56% reduction in progression or death with RT + ICI, whereas cohorts relying on lower-dose palliative regimens demonstrated a less pronounced benefit (27, 30). Clinical trials such as PEMBRO-RT further support this concept, showing that SBRT can induce abscopal-type responses and improve outcomes when combined with anti-PD-1 therapy in metastatic NSCLC, reinforcing the systemic immune-amplifying capacity of high-dose RT (43, 44).

Consistent with these data, high-dose hypofractionated RT was the predominant approach across the included studies and likely contributed significantly to the magnitude of observed benefit. Among the six studies, 78% of irradiated lesions were treated with SBRT/SABR (typically BED ≥ 100 Gy_1 0_), whereas conventional regimens such as 8 Gy × 1 or 20 Gy × 5 were used in only a minority of cases. This treatment pattern parallels the favorable survival outcomes in our meta-analysis, in which RT + ICI reduced the risk of death by 42% (pooled HR 0.58) and the risk of progression or death by 56% (HR 0.44) compared with ICI alone. Exploratory analyses also showed that cohorts using lower-dose, protracted schedules or delivering RT more than 4 weeks after starting ICIs exhibited attenuated survival gains. Together, these results suggest that early, high-dose focal irradiation is the most immunogenically and clinically effective strategy when integrating RT with ICIs in patients with NSCLC with bone metastases.

The biological advantages of high-dose regimens have been well characterized. Preclinical studies demonstrate that ablative radiation more effectively induces ICD, activates type I interferon signaling, and expands diverse tumor-specific T-cell clones, thereby amplifying ICI-mediated immune priming (35, 41, 42). Clinically, hypofractionated SBRT to a limited number of lesions has been associated with higher out-of-field response rates and more durable systemic control when combined with PD-1/PD-L1 inhibitors than either modality alone (43–45). In pooled analyses of the PEMBRO-RT and MDACC randomized trials in metastatic NSCLC, adding SBRT to pembrolizumab nearly doubled abscopal-type response rates (19.7% vs 41.7%) and prolonged median PFS (4.4 to 9.0 months) and OS (8.7 to 19.2 months) without new safety concerns (43, 45). The magnitude of benefit in these trials closely mirrors our pooled estimates (HR 0.58 for OS and HR 0.44 for PFS), indicating that the survival gains in our bone-metastatic cohort likely reflect a broader immunoradiotherapy effect observed across metastatic NSCLC. Similar findings have emerged from multiple Phase I/II trials integrating SBRT with PD-1/PD-L1 blockade, particularly when RT is delivered in close temporal proximity to immunotherapy (44, 45).

Our results extend these observations to patients with NSCLC with bone metastases, a population historically under-represented in prospective ICI trials and known to experience poorer survival and higher SRE rates with immunotherapy alone (5, 15, 29–31). In real-world ICI-treated cohorts, bone metastases and SREs are associated with substantially worse outcomes, with SREs tripling the risk of death in some reports (5, 30, 31). Against this unfavorable clinical backdrop, the marked reduction in SREs in our analysis—one SRE in the RT + ICI arm vs 25 in the ICI-alone arm (pooled OR = 0.16)—is consistent with the durable local control typically achieved with high-dose SBRT/SABR in weight-bearing bones and exceeds expectations from systemic therapy alone (1, 6, 17, 27, 29). Nevertheless, the apparent SRE reduction is driven by sparse events in a small number of cohorts and should be interpreted cautiously pending confirmation in adequately powered prospective studies. At the same time, the improvements in OS and PFS suggest that high-dose RT in this setting is not purely palliative but functions as a potent immunologic enhancer. Thus, in patients with NSCLC with bone metastases—who are at high risk of early skeletal complications and rapid systemic progression—early integration of high-dose hypofractionated RT with ICIs may stabilize the metastatic skeleton, reduce SRE burden, and optimize systemic anti-tumor immunity.

Another critical factor is the timing of RT relative to ICI administration. Approximately 80% of patients in the RT + ICI arms of the included studies received RT either before or concurrently with ICI initiation, and in this context, we observed significant improvements in both OS and PFS compared with ICI alone. By contrast, the largest real-world cohort, in which RT was frequently delivered > 4 weeks after starting ICIs, showed the weakest survival signal (HR for OS ≈ 0.83) and accounted for most of the between-study heterogeneity. Excluding this cohort eliminated statistical heterogeneity and strengthened the pooled OS estimate (HR 0.52, 95% CI 0.42–0.63). These findings indicate that sequencing is a key modifier of treatment effect and that delaying RT reduces the synergistic interactions underlying the clinical advantage of RT + ICI. For patients with bone metastases, who are particularly vulnerable to SREs and symptomatic decline, timely initiation of local RT within ICI-based regimens may not only relieve symptoms but also maximize the systemic immunologic benefits of checkpoint blockade, offering a strategy that addresses both local and systemic disease burdens (44, 46–48).

Reassuringly, clinical evidence from other metastatic settings aligns with the observed early-integration signal. In patients with NSCLC with brain metastases, Yu et al. reported (49) that initiating RT without delay and combining it with immediate PD-1/PD-L1 blockade was associated with superior intracranial PFS and OS compared with approaches in which RT was deferred, supporting the benefit of a short RT–ICI interval. Similarly, Antelo et al. found (50) that, across patients with brain metastases from various primary tumors, focal stereotactic RT delivered concurrently with ICIs or within 30 days of ICI initiation yielded the most favorable intracranial PFS without increasing neurotoxicity, whereas longer intervals were associated with less favorable outcomes. An international individual-patient meta-analysis by Lehrer et al. (51) further demonstrated that concurrent stereotactic radiosurgery (SRS) and ICIs improved 1-year survival and regional brain control compared with non-concurrent sequencing, without a clinically meaningful increase in radionecrosis. Collectively, these data reinforce the concept that tighter temporal coupling of RT and immunotherapy may translate into improved clinical outcomes.

From a mechanistic standpoint, these findings are biologically plausible. Early or concurrent RT with ICI is thought to maximize the immunologic ‘priming window’, during which radiation-induced antigen release, type I interferon signaling, and dendritic-cell activation coincide with pharmacologic reversal of T-cell exhaustion mediated by PD-1/PD-L1 or CTLA-4 blockade (44, 52). Administering RT while effector T cells are expanding and being reinvigorated may promote the development of a broader and more durable tumor-specific T-cell repertoire, whereas delayed RT given after the peak of immune activation may function primarily as a local cytotoxic therapy with diminished systemic impact. This concept aligns with the ‘window-of-opportunity’ hypothesis, in which early RT acts as a focused antigenic boost that capitalizes on ICI-induced immune activation to generate a stronger systemic anti-tumor response.

In this context, timing considerations may be especially relevant for patients with NSCLC and bone metastases who are at high risk for early SREs and rapid systemic progression. In our pooled analysis, the favorable survival outcomes and marked reduction in SREs observed with RT + ICI are consistent with a model in which early integration of RT not only stabilizes vulnerable skeletal lesions but also contributes to systemic immune control. Although our dataset does not allow precise definition of an optimal RT–ICI interval, the diminished benefit observed when RT was administered more than 4 weeks after ICI initiation, together with consistent findings from other metastatic settings, supports the hypothesis that RT should ideally be delivered before or within a narrow window surrounding ICI commencement. This warrants further prospective evaluation of RT timing strategies in this high-risk population.

The consistent survival benefit, preserved safety profile, and substantial reduction in SREs observed in our meta-analysis carry important implications for the clinical management of patients with NSCLC with bone metastases. Historically, bone disease in this setting has been treated primarily as a source of pain or mechanical instability, with RT administered in a largely palliative, symptom-driven manner while systemic therapy decisions were guided predominantly by extra-osseous disease burden. Our findings, supported by emerging prospective and real-world evidence, suggest that this separation between local and systemic care may underestimate the value of bone-directed RT when integrated with ICIs (26–30).

In the FORCE Phase II trial, adding palliative RT to PD-1 blockade in advanced NSCLC was feasible and showed signals of improved disease control and survival without excess toxicity (26), and several retrospective studies have similarly reported that prior or concurrent RT does not compromise the safety of nivolumab-based immunotherapy and may be associated with improved outcomes (51). More specifically, studies focusing on NSCLC with bone metastases have shown that combining ICIs with local RT delivered to bone lesions can induce abscopal responses and improve PFS and OS compared with immunotherapy alone (27–30). These findings are consistent with our pooled results, in which RT + ICI reduced the risk of death by 42%, halved the risk of progression or death, and decreased the SRE burden from 25 events in the ICI-alone arm to a single event in the RT + ICI group.

From a practical perspective, these converging data support a more proactive and integrated use of bone-directed RT in the immunotherapy era. Rather than reserving RT solely for uncontrolled pain or impending fracture, it appears reasonable to consider early, high-quality RT delivery to dominant or high-risk bone lesions as part of the initial ICI-based treatment strategy for appropriately selected patients with NSCLC and radiologically confirmed bone metastases, particularly those with axial or weight-bearing lesions at high risk of SREs (27–30). Notably, across prospective and real-world cohorts, RT + ICIs has generally been well tolerated, with no substantial increase in severe immune- or radiation-related toxicity when delivered within a multidisciplinary framework and with careful patient selection (26, 28, 31). Nevertheless, the observational design and heterogeneity of existing studies highlight the need for dedicated prospective trials in patients with NSCLC with bone metastases to validate the magnitude of survival and SRE benefit and refine patient selection and RT parameters.

Beyond these clinical and biological considerations, our study has several important strengths. First, we adhered to rigorous scientific standards, following PRISMA 2020 guidelines and prospectively registering our protocol in PROSPERO. Our comprehensive search across PubMed, Web of Science, Scopus, Embase, and the Cochrane Library ensured the inclusion of the most relevant and up-to-date studies. By integrating data from six high-quality cohort studies encompassing 1,631 patients, we achieved a robust sample size that enhances the reliability and generalizability of our findings and provides sufficient statistical power to detect moderate effect sizes. Second, this study also offers a comprehensive evaluation of the impact of RT + ICIs on OS, PFS, ORR, and SREs in patients with NSCLC with bone metastases. The combination therapy demonstrated significant survival and skeletal benefits while maintaining a safety profile comparable to ICI alone. Finally, through sensitivity analyses and subgroup assessments, we identified potential effect modifiers, including RT timing and dose intensity, which provide valuable direction for future research and clinical practice.

Despite these strengths, certain limitations must be acknowledged. First, because the analysis included five retrospective and one prospective cohort study, there is inherent susceptibility to selection bias and residual confounding, including baseline performance status and treatment-selection bias. Radiotherapy was not randomly assigned in most studies, and patients selected for RT plus ICI may have had more favorable baseline characteristics, such as limited metastatic burden or better performance status, which could partially bias survival outcomes. Second, substantial heterogeneity in OS and ORR, driven largely by a single large real-world study, may limit the robustness of our pooled estimates, although sensitivity analyses mitigated this issue. Therefore, the pooled OS and ORR estimates should be interpreted with caution. Third, the limited number of studies prevented formal meta-regression, meaning that our exploration of effect modifiers remains hypothesis-generating rather than definitive. Fourth, the small number of included studies and the marked imbalance in study size limit the interpretability of funnel plots; small-study effects and publication bias cannot be ruled out. Additionally, reporting of concomitant bone-targeted agents such as zoledronic acid or denosumab was incomplete across studies, preventing formal adjustment for their potential contributions to SRE reduction or survival, and representing an additional source of residual confounding. Finally, SRE estimates were derived from only four studies with sparse events, including a single event in the RT + ICI arm, which may overstate the effect size and limit certainty; accordingly, absolute-effect translations (e.g., ARR/NNT) may not be reliable in this setting. In addition, variability in SRE definitions and reporting may have influenced skeletal outcomes. To improve comparability and reduce uncertainty in future studies, key RT and assessment parameters should be prespecified and consistently reported, including RT intent and target definition, dose–fractionation with BED, RT–ICI sequencing, and standardized criteria for response and SREs.

In conclusion, low- to moderate-quality observational evidence suggests that RT + ICIs may provide clinical benefit for NSCLC patients with bone metastases, improving both OS and PFS without increasing severe toxicity. These findings support further evaluation of incorporating RT into immunotherapy-based strategies in this high-risk population. Notably, SRE estimates were based on few studies with very low event counts, which limits precision and may inflate the apparent effect size. From a clinical perspective, early incorporation of high-dose hypofractionated RT (SBRT/SABR), when feasible, to dominant and/or symptomatic high-risk bone lesions may be considered in selected patients within a multidisciplinary ICI-based treatment plan. However, ORR remained heterogeneous across studies and should be interpreted cautiously. Given the largely observational evidence base, prospective phase III randomized trials are needed to confirm causality and refine patient selection.

## References

[1] Xue M Ma L Zhang P Yang H Wang Z . New insights into non-small cell lung cancer bone metastasis: mechanisms and therapies. Int J Biol Sci. (2024) 20:5747–63. doi: 10.7150/ijbs.100960, PMID: 39494330 PMC11528464

[2] Del Conte A De Carlo E Bertoli E Stanzione B Revelant A Bertola M . Bone Metastasis and Immune Checkpoint Inhibitors in Non-Small Cell Lung Cancer (NSCLC): Microenvironment and Possible Clinical Implications. Int J Mol Sci. (2022) 23:6832. doi: 10.3390/ijms23126832, PMID: 35743275 PMC9224636

[3] Cao Z Zheng R Li J Wang X Ding C Zhang F . Risk factors of bone metastasis in lung adenocarcinoma. BMC Pulm Med. (2025) 25:299. doi: 10.1186/s12890-025-03702-0, PMID: 40604601 PMC12224695

[4] Knapp BJ Devarakonda S Govindan R . Bone metastases in non-small cell lung cancer: a narrative review. J Thorac Dis. (2022) 14:1696–712. doi: 10.21037/jtd-21-1502, PMID: 35693589 PMC9186248

[5] Qin A Zhao S Miah A Wei L Patel S Johns A . Bone Metastases, Skeletal-Related Events, and Survival in Patients With Metastatic Non-Small Cell Lung Cancer Treated With Immune Checkpoint Inhibitors. J Natl Compr Canc Netw. (2021) 19:915–21. doi: 10.6004/jnccn.2020.7668, PMID: 33878726 PMC8752085

[6] Coleman R Hadji P Body JJ Santini D Chow E Terpos E . Electronic address: clinicalguidelines@esmo.org. Bone health in cancer: ESMO Clinical Practice Guidelines. Ann Oncol. (2020) 31:1650–63. doi: 10.1016/j.annonc.2020.07.019, PMID: 32801018

[7] Gandhi L Rodríguez-Abreu D Gadgeel S Esteban E Felip E De Angelis F . Pembrolizumab plus Chemotherapy in Metastatic Non-Small-Cell Lung Cancer. N Engl J Med. (2018) 378:2078–92. doi: 10.1056/NEJMoa1801005, PMID: 29658856

[8] West H McCleod M Hussein M Morabito A Rittmeyer A Conter HJ . Atezolizumab in combination with carboplatin plus nab-paclitaxel chemotherapy compared with chemotherapy alone as first-line treatment for metastatic non-squamous non-small-cell lung cancer (IMpower130): a multicentre, randomised, open-label, phase 3 trial. Lancet Oncol. (2019) 20:924–37. doi: 10.1016/S1470-2045(19)30167-6, PMID: 31122901

[9] Reck M Rodríguez-Abreu D Robinson AG Hui R Csőszi T Fülöp A . Five-Year Outcomes With Pembrolizumab Versus Chemotherapy for Metastatic Non-Small-Cell Lung Cancer With PD-L1 Tumor Proportion Score ≥ 50. J Clin Oncol. (2021) 39:2339–49. doi: 10.1200/JCO.21.00174, PMID: 33872070 PMC8280089

[10] Sun L Bleiberg B Hwang WT Marmarelis ME Langer CJ Singh A . Association Between Duration of Immunotherapy and Overall Survival in Advanced Non-Small Cell Lung Cancer. JAMA Oncol. (2023) 9:1075–82. doi: 10.1001/jamaoncol.2023.1891, PMID: 37270700 PMC10240399

[11] Guo S Yao Y Tang Y Xin Z Wu D Ni C . Radiation-induced tumor immune microenvironments and potential targets for combination therapy. Signal Transduct Target Ther. (2023) 8:205. doi: 10.1038/s41392-023-01462-z, PMID: 37208386 PMC10199044

[12] Zhu S Wang Y Tang J Cao M . Radiotherapy induced immunogenic cell death by remodeling tumor immune microenvironment. Front Immunol. (2022) 13:1074477. doi: 10.3389/fimmu.2022.1074477, PMID: 36532071 PMC9753984

[13] Zhu YJ Chang XS Zhou R Chen YD Ma HC Xiao ZZ . Bone metastasis attenuates efficacy of immune checkpoint inhibitors and displays "cold" immune characteristics in Non-small cell lung cancer. Lung Cancer. (2022) 166:189–96. doi: 10.1016/j.lungcan.2022.03.006, PMID: 35306320

[14] Zhu Y She J Sun R Yan X Huang X Wang P . Impact of bone metastasis on prognosis in non-small cell lung cancer patients treated with immune checkpoint inhibitors: a systematic review and meta-analysis. Front Immunol. (2024) 15:1493773. doi: 10.3389/fimmu.2024.1493773, PMID: 39575263 PMC11578953

[15] Landi L D'Incà F Gelibter A Chiari R Grossi F Delmonte A . Bone metastases and immunotherapy in patients with advanced non-small-cell lung cancer. J Immunother Cancer. (2019) 7:316. doi: 10.1186/s40425-019-0793-8, PMID: 31752994 PMC6868703

[16] Lim AR Yoon WS Park S Rim CH . Systematic Review-Based Treatment Algorithm for the Multidisciplinary Treatment of Lung Cancer Bone Metastases. Cancers (Basel). (2024) 16:4144. doi: 10.3390/cancers16244144, PMID: 39766043 PMC11674356

[17] Dong H Lan A Gao J An Y Chu L Yang X . Prognostic significance of bone metastasis and clinical value of bone radiotherapy in metastatic non-small cell lung cancer receiving PD-1/PD-L1 inhibitors: results from a multicenter, prospective, observational study. Transl Lung Cancer Res. (2024) 13:2603–16. doi: 10.21037/tlcr-24-441, PMID: 39507037 PMC11535830

[18] Jiang J Li H Ma Q Liu J Ren F Song Y . Synergies between radiotherapy and immunotherapy: a systematic review from mechanism to clinical application. Front Immunol. (2025) 16:1554499. doi: 10.3389/fimmu.2025.1554499, PMID: 40861450 PMC12375553

[19] Liu S Wang W Hu S Jia B Tuo B Sun H . Radiotherapy remodels the tumor microenvironment for enhancing immunotherapeutic sensitivity. Cell Death Dis. (2023) 14:679. doi: 10.1038/s41419-023-06211-2, PMID: 37833255 PMC10575861

[20] Wang L Lynch C Pitroda SP Piffkó A Yang K Huser AK . Radiotherapy and immunology. J Exp Med. (2024) 221:e20232101. doi: 10.1084/jem.20232101, PMID: 38771260 PMC11110906

[21] Zhang Z Liu X Chen D Yu J . Radiotherapy combined with immunotherapy: the dawn of cancer treatment. Signal Transduct Target Ther. (2022) 7:258. doi: 10.1038/s41392-022-01102-y, PMID: 35906199 PMC9338328

[22] Jongbloed M Bartolomeo V Bortolot M Darwesh S Huijs JWJ Dursun S . Impact of Immune Checkpoint Inhibitors and Local Radical Treatment on Survival Outcomes in Synchronous Oligometastatic NSCLC. JTO Clin Res Rep. (2025) 6:100790. doi: 10.1016/j.jtocrr.2025.100790, PMID: 39990139 PMC11847110

[23] Page MJ McKenzie JE Bossuyt PM Boutron I Hofmann TC Mulrow CD . The PRISMA 2020 statement: an updated guideline for reporting systematic reviews. BMJ. (2021) 372:n71. doi: 10.1136/bmj.n71, PMID: 33782057 PMC8005924

[24] Higgins JPT Thomas J Chandler J Cumpston M Li T Page MJ . Cochrane handbook for systematic reviews of interventions version 6.3. Chichester: Cochrane (2022). Available online at: www.training.cochrane.org/handbook (Accessed February 11, 2026).

[25] Palma DA Senan S Tsujino K Barriger RB Rengan R Moreno M . Predicting radiation pneumonitis after chemoradiation therapy for lung cancer: an international individual patient data meta-analysis. Int J Radiat Oncol Biol Phys. (2013) 85:444–50. doi: 10.1016/j.ijrobp.2012.04.043, PMID: 22682812 PMC3448004

[26] Bozorgmehr F Chung I Fischer JR Bischof M Atmaca A Wetzel S . Reconsidering palliative radiotherapy in addition to PD-1 blockade for non-small cell lung cancer: results from the FORCE phase II trial (AIO/YMO-TRK-0415). Clin Exp Metastasis. (2025) 42:42. doi: 10.1007/s10585-025-10358-x, PMID: 40702361 PMC12287132

[27] Asano Y Hayashi K Miwa S Taniguchi Y Okuda M Matsumoto I . Combination of immune checkpoint inhibitors and radiotherapy for bone metastases induces an abscopal effect and improves outcomes in non-small cell lung cancer. Oncol Lett. (2025) 30:524. doi: 10.3892/ol.2025.15270, PMID: 40995141 PMC12455549

[28] Beyon J Collins JE Welch CA Kamran A . Immunotherapy with and without radiotherapy following the diagnosis of bone metastasis for stage IV non-small cell carcinoma. J Cancer Res Clin Oncol. (2025) 151:309. doi: 10.1007/s00432-025-06303-w, PMID: 41165912 PMC12575909

[29] Facilissimo I Natoli G Gaspari F Comandone T Bongiovanni D Gollini P . The role of bone radiotherapy during immune checkpoint inhibitors treatment of non-small-cell lung cancer: a single-institution experience. Ther Adv Med Oncol. (2025) 17:17588359251332451. doi: 10.1177/17588359251332451, PMID: 40336632 PMC12056327

[30] Qiang H Lei Y Shen Y Li J Zhong H Zhong R . Pembrolizumab monotherapy or combination therapy for bone metastases in advanced non-small cell lung cancer: a real-world retrospective study. Transl Lung Cancer Res. (2022) 11:87–99. doi: 10.21037/tlcr-21-1033, PMID: 35242630 PMC8825649

[31] Ratnayake G Shanker M Roberts K Mason R Hughes BGM Lwin Z . Prior or concurrent radiotherapy and nivolumab immunotherapy in non-small cell lung cancer. Asia Pac J Clin Oncol. (2020) 16:56–62. doi: 10.1111/ajco.13242, PMID: 31721446

[32] Fucikova J Kepp O Kasikova L Petroni G Yamazaki T Liu P . Detection of immunogenic cell death and its relevance for cancer therapy. Cell Death Dis. (2020) 11:1013. doi: 10.1038/s41419-020-03221-2, PMID: 33243969 PMC7691519

[33] Qi H Li Y Geng Y Wan X Cai X . Nanoparticle-mediated immunogenic cell death for cancer immunotherapy. Int J Pharm. (2024) 656:124045. doi: 10.1016/j.ijpharm.2024.124045, PMID: 38561134

[34] Amiri M Molavi O Sabetkam S Jafari S Montazersaheb S . Stimulators of immunogenic cell death for cancer therapy: focusing on natural compounds. Cancer Cell Int. (2023) 23:200. doi: 10.1186/s12935-023-03058-7, PMID: 37705051 PMC10500939

[35] Zhu M Yang M Zhang J Yin Y Fan X Zhang Y . Immunogenic Cell Death Induction by Ionizing Radiation. Front Immunol. (2021) 12:705361. doi: 10.3389/fimmu.2021.705361, PMID: 34489957 PMC8417736

[36] Cheng CC Chang J Ho AS Sie ZL Peng CL Wang CL . Tumor-intrinsic IFNα and CXCL10 are critical for immunotherapeutic efficacy by recruiting and activating T lymphocytes in tumor microenvironment. Cancer Immunol Immunother. (2024) 73:175. doi: 10.1007/s00262-024-03761-y, PMID: 38953994 PMC11219622

[37] Lim RJ Salehi-Rad R Tran LM Oh MS Dumitras C Crosson WP . CXCL9/10-engineered dendritic cells promote T cell activation and enhance immune checkpoint blockade for lung cancer. Cell Rep Med. (2024) 5:101479. doi: 10.1016/j.xcrm.2024.101479, PMID: 38518770 PMC11031384

[38] Wang CL Ho AS Chang CC Sie ZL Peng CL Chang J . Radiotherapy enhances CXCR3highCD8+ T cell activation through inducing IFNγ-mediated CXCL10 and ICAM-1 expression in lung cancer cells. Cancer Immunol Immunother. (2023) 72:1865–80. doi: 10.1007/s00262-023-03379-6, PMID: 36688994 PMC10198930

[39] Bergerud KMB Berkseth M Pardoll DM Ganguly S Kleinberg LR Lawrence J . Radiation Therapy and Myeloid-Derived Suppressor Cells: Breaking Down Their Cancerous Partnership. Int J Radiat Oncol Biol Phys. (2024) 119:42–55. doi: 10.1016/j.ijrobp.2023.11.050, PMID: 38042450 PMC11082936

[40] Lukas L Zhang H Cheng K Epstein A . Immune Priming with Spatially Fractionated Radiation Therapy. Curr Oncol Rep. (2023) 25:1483–96. doi: 10.1007/s11912-023-01473-7, PMID: 37979032 PMC10728252

[41] Rodriguez-Ruiz ME Vitale I Harrington KJ Melero I Galluzzi L . Immunological impact of cell death signaling driven by radiation on the tumor microenvironment. Nat Immunol. (2020) 21:120–34. doi: 10.1038/s41590-019-0561-4, PMID: 31873291

[42] Chow J Hoffend NC Abrams SI Schwaab T Singh AK Muhitch JB . Radiation induces dynamic changes to the T cell repertoire in renal cell carcinoma patients. Proc Natl Acad Sci U S A. (2020) 117:23721–9. doi: 10.1073/pnas.2001933117, PMID: 32900949 PMC7519245

[43] Theelen WSME Peulen HMU Lalezari F van der Noort V de Vries JF Aerts JGJV . Effect of Pembrolizumab After Stereotactic Body Radiotherapy vs Pembrolizumab Alone on Tumor Response in Patients With Advanced Non-Small Cell Lung Cancer: Results of the PEMBRO-RT Phase 2 Randomized Clinical Trial. JAMA Oncol. (2019) 5:1276–82. doi: 10.1001/jamaoncol.2019.1478, PMID: 31294749 PMC6624814

[44] Huang J Theelen WSME Belcaid Z Najjar M van der Geest D Singh D . Combination of pembrolizumab and radiotherapy induces systemic antitumor immune responses in immunologically cold non-small cell lung cancer. Nat Cancer. (2025) 6:1676–92. doi: 10.1038/s43018-025-01018-w, PMID: 40696153 PMC12559004

[45] Theelen WSME Chen D Verma V Hobbs BP Peulen HMU Aerts JGJV . Pembrolizumab with or without radiotherapy for metastatic non-small-cell lung cancer: a pooled analysis of two randomised trials. Lancet Respir Med. (2021) 9:467–75. doi: 10.1016/S2213-2600(20)30391-X, PMID: 33096027

[46] Tian W Chu X Tanzhu G Zhou R . Optimal timing and sequence of combining stereotactic radiosurgery with immune checkpoint inhibitors in treating brain metastases: clinical evidence and mechanistic basis. J Transl Med. (2023) 21:244. doi: 10.1186/s12967-023-04089-4, PMID: 37020242 PMC10077682

[47] Jokimäki A Hietala H Lemma J Karhapää H Rintala A Kaikkonen JP . Previous radiotherapy improves treatment responses and causes a trend toward longer time to progression among patients with immune checkpoint inhibitor-related adverse events. Cancer Immunol Immunother. (2023) 72:3337–47. doi: 10.1007/s00262-023-03494-4, PMID: 37486396 PMC10491510

[48] Verma S Young S Boldt G Blanchette P Lock M Helou J . Immunotherapy and Radiation Therapy Sequencing in Breast Cancer: A Systematic Review. Int J Radiat Oncol Biol Phys. (2024) 118:1422–34. doi: 10.1016/j.ijrobp.2024.01.001, PMID: 38195030

[49] Yu Y Chen H Tian Z Zhang Q Shui Y Shen L . Improved survival outcome with not-delayed radiotherapy and immediate PD-1/PD-L1 inhibitor for non-small-cell lung cancer patients with brain metastases. J Neurooncol. (2023) 165:127–37. doi: 10.1007/s11060-023-04459-4, PMID: 37848757 PMC10638122

[50] Antelo G Comas S Casas F Valduvieco I Barreto T Laplana M . Clinical outcomes and timing on the combination of focal radiation therapy and immunotherapy for the treatment of brain metastases. Front Immunol. (2023) 14:1236398. doi: 10.3389/fimmu.2023.1236398, PMID: 37915576 PMC10616465

[51] Lehrer EJ Peterson J Brown PD Sheehan JP Quiñones-Hinojosa A Zaorsky NG . Treatment of brain metastases with stereotactic radiosurgery and immune checkpoint inhibitors: An international meta-analysis of individual patient data. Radiother Oncol. (2019) 130:104–12. doi: 10.1016/j.radonc.2018.08.025, PMID: 30241791

[52] Aliru ML Schoenhals JE Venkatesulu BP Anderson CC Barsoumian HB Younes AI . Radiation therapy and immunotherapy: what is the optimal timing or sequencing? Immunotherapy. (2018) 10:299–316. doi: 10.2217/imt-2017-0082, PMID: 29421979 PMC5810851

