# Supplementary material for: Radiotherapy plus immune checkpoint inhibitors versus immune checkpoint inhibitors alone for non-small cell lung cancer with bone metastases: a systematic review and meta-analysis of comparative cohort studies
Source: Front Immunol. 2026 Feb 19;17:1773998. doi: 10.3389/fimmu.2026.1773998 (PMC12960617; doi:10.3389/fimmu.2026.1773998)
Supplement: Supplementary file 1 [file Table1.docx]

### Supplementary Methods: Literature search strategy

We systematically searched PubMed, Web of Science Core Collection, Scopus, Embase and the Cochrane Library for studies published from **January 2010 to October 2025**. No language restrictions other than English were applied. The detailed search strategies for each database are listed below.

#### 1. PubMed

**Database:** PubMed (MEDLINE)
**Date range:** 2010-01-01 to 2025-10-31
**Search fields:** MeSH terms and Title/Abstract

("Carcinoma, Non-Small-Cell Lung"[Mesh] OR (NSCLC[tiab] OR "non small cell lung"[tiab] OR "non-small cell lung"[tiab] OR "lung adenocarcinoma"[tiab] OR "lung squamous cell carcinoma"[tiab])) AND ("Bone Neoplasms"[Mesh] OR "Bone Metastases"[Mesh] OR ("bone metastas*"[tiab] OR "skeletal metastas*"[tiab] OR "spinal metastas*"[tiab] OR "vertebral metastas*"[tiab] OR "osseous metastas*"[tiab] OR "bone lesion*"[tiab] OR "bone only disease"[tiab] OR oligometastas*[tiab] OR ((bone[tiab] OR skeletal[tiab] OR osseous[tiab] OR spine[tiab] OR vertebral[tiab] OR vertebrae[tiab] OR rib[tiab] OR pelvic[tiab] OR iliac[tiab] OR femoral[tiab] OR humeral[tiab] OR skull[tiab]) AND (metastas*[tiab] OR secondar*[tiab] OR lesion*[tiab]))) AND ("Radiotherapy"[Mesh] OR "Stereotactic Body Radiotherapy"[Mesh] OR "Radiosurgery"[Mesh] OR radiotherap*[tiab] OR irradiation[tiab] OR RT[tiab] OR SBRT[tiab] OR SABR[tiab] OR "Stereotactic Ablative Radiotherapy"[tiab] OR "Stereotactic Body Radiation Therapy"[tiab] OR IMRT[tiab] OR "Intensity Modulated Radiation Therapy"[tiab] OR radiosurgery[tiab] OR "gamma knife"[tiab] OR "cyberknife"[tiab] OR "palliative radiation"[tiab] OR "definitive radiation"[tiab] OR "local therapy"[tiab] OR "local ablative therapy"[tiab])

AND ("Immunotherapy"[Mesh] OR "Immune Checkpoint Inhibitors"[Mesh] OR immunotherap*[tiab] OR "immune checkpoint"[tiab] OR ICI[tiab] OR "checkpoint inhibitor*"[tiab] OR "checkpoint block*"[tiab] OR "PD-1"[tiab] OR "programmed cell death 1"[tiab] OR "anti-PD-1"[tiab] OR "PD-L1"[tiab] OR "programmed death ligand 1"[tiab] OR "anti-PD-L1"[tiab] OR "CTLA-4"[tiab] OR "cytotoxic T-lymphocyte antigen 4"[tiab] OR "anti-CTLA-4"[tiab] OR pembrolizumab[tiab] OR nivolumab[tiab] OR atezolizumab[tiab] OR durvalumab[tiab] OR ipilimumab[tiab] OR camrelizumab[tiab] OR toripalimab[tiab] OR tislelizumab[tiab] OR sintilimab[tiab] OR avelumab[tiab] OR cemiplimab[tiab] OR dostarlimab[tiab]) AND

("2010/01/01"[Date - Publication] : "2025/10/31"[Date - Publication])

#### 2. Web of Science Core Collection

**Database:** Web of Science Core Collection
**Search field:** Topic (TS)
**Search date:** up to 31 October 2025

**Step 1 – NSCLC with bone involvement (#1)**

TS=( (("non small cell lung" OR "non-small cell lung" OR NSCLC OR "lung adenocarcinoma" OR "lung squamous cell carcinoma") AND (bone OR skeletal OR osseous OR spine OR vertebral OR vertebrae OR "skeletal-related" OR oligometastas*) AND (metasta* OR secondar* OR lesion* OR progression) ) OR ("bone metastas*" OR "skeletal metastas*" OR "osseous metastas*" OR "bone lesion*" OR "spinal metastas*" OR "vertebral metastas*" OR "bone only disease") )

**Step 2 – Radiotherapy concept (#2)**

TS=( radiotherap* OR radiotherapy OR irradiation OR "radiation therapy" OR RT OR SBRT OR SABR OR "Stereotactic Ablative Radiotherapy" OR "Stereotactic Body Radiation Therapy" OR IMRT OR "Intensity Modulated Radiation Therapy" OR radiosurgery OR "gamma knife" OR "cyberknife" OR "palliative radiation" OR "definitive radiation" OR "local therapy" OR "local ablative therapy")

**Step 3 – Immunotherapy / ICI concept (#3)**

TS=( immunotherap* OR "immune checkpoint" OR ICI OR "checkpoint inhibitor*" OR "checkpoint block*" OR "PD-1" OR "programmed cell death 1" OR "PD 1" OR "anti-PD-1" OR "anti PD 1" OR "PD-L1" OR "programmed death ligand 1" OR "PD L1" OR "anti-PD-L1" OR "anti PD L1" OR "CTLA-4" OR "cytotoxic T-lymphocyte antigen 4" OR "CTLA 4" OR "anti-CTLA-4" OR "anti CTLA 4" OR pembrolizumab OR nivolumab OR atezolizumab OR durvalumab OR camrelizumab OR toripalimab OR tislelizumab OR sintilimab OR ipilimumab OR avelumab OR cemiplimab OR dostarlimab OR "immune-oncolog*" OR immuno-oncolog* OR "anti-PD1" OR "anti-PDL1" )

**Final combination:**

#1 AND #2 AND #3

#### 3. Scopus

**Database:** Scopus
**Search date:** up to 31 October 2025

**Block 1 – NSCLC with bone involvement (Search 1#):**

( ( ("non small cell lung" OR "non-small cell lung" OR NSCLC OR "lung adenocarcinoma" OR "lung squamous cell carcinoma") AND (bone OR skeletal OR osseous OR spine OR vertebral OR vertebrae OR "skeletal-related" OR oligometastas*) AND (metastas* OR secondar* OR lesion* OR progression) ) OR ("bone metastas*" OR "skeletal metastas*" OR "osseous metastas*" OR "bone lesion*" OR "spinal metastas*" OR "vertebral metastas*" OR "bone only disease"))

**Block 2 – Radiotherapy (Search 2#):**

(radiotherap* OR radiotherapy OR irradiation OR "radiation therapy" OR RT OR SBRT OR SABR OR "Stereotactic Ablative Radiotherapy" OR "Stereotactic Body Radiation Therapy" OR IMRT OR "Intensity Modulated Radiation Therapy" OR radiosurgery OR "gamma knife" OR "cyberknife" OR "palliative radiation" OR "definitive radiation" OR "local therapy" OR "local ablative therapy")

**Block 3 – Immunotherapy / ICI (Search 3#):**

(immunotherap* OR "immune checkpoint" OR ICI OR "checkpoint inhibitor*" OR "checkpoint block*" OR "PD-1" OR "programmed cell death 1" OR "PD 1" OR "anti-PD-1" OR "anti PD 1" OR "PD-L1" OR "programmed death ligand 1" OR "PD L1" OR "anti-PD-L1" OR "anti PD L1" OR "CTLA-4" OR "cytotoxic T-lymphocyte antigen 4" OR "CTLA 4" OR "anti-CTLA-4" OR "anti CTLA 4" OR pembrolizumab OR nivolumab OR atezolizumab OR durvalumab OR camrelizumab OR toripalimab OR tislelizumab OR sintilimab OR ipilimumab OR avelumab OR cemiplimab OR dostarlimab OR "immune-oncolog*" OR immuno-oncolog* OR "anti-PD1" OR "anti-PDL1")

**Final combination in Scopus:**

1# AND 2# AND 3#

#### 4. Embase

**Database:** Embase (via Ovid or Elsevier)
**Search date:** up to 31 October 2025

('non small cell lung cancer'/exp OR 'non small cell lung cancer' OR 'NSCLC' OR 'non-small cell lung' OR 'lung adenocarcinoma' OR 'lung squamous cell carcinoma')

AND ('bone metastasis'/exp OR 'bone metastasis' OR 'bone metastases' OR 'skeletal metastasis' OR 'skeletal metastases' OR 'bone lesion*' OR 'spinal metastasis' OR 'vertebral metastasis' OR 'osseous metastasis' OR ((bone OR skeletal OR spine OR vertebral OR osseous) AND (metastas* OR secondar* OR lesion*))) AND ('radiotherapy'/exp OR 'stereotactic body radiotherapy'/exp OR radiotherap* OR radiotherapy OR irradiation OR 'radiation therapy' OR RT OR SBRT OR SABR OR 'stereotactic ablative radiotherapy' OR 'stereotactic body radiation therapy' OR 'intensity modulated radiation therapy' OR IMRT OR radiosurgery OR 'gamma knife' OR 'cyberknife' OR 'palliative radiation' OR 'definitive radiation' OR 'local therapy' OR 'local ablative therapy') AND ('immunotherapy'/exp OR 'immune checkpoint inhibitor'/exp OR 'pd-1 inhibitor'/exp OR 'pd-l1 inhibitor'/exp OR 'ctla-4 inhibitor'/exp

OR immunotherap* OR 'immune checkpoint' OR ICI OR 'checkpoint inhibitor*' OR 'checkpoint block*' OR 'pd-1' OR 'pd l1' OR 'programmed cell death 1' OR 'programmed death ligand 1' OR 'CTLA-4' OR 'cytotoxic T-lymphocyte antigen 4' OR 'anti-pd-1' OR 'anti-pd-l1' OR 'anti-ctla-4' OR pembrolizumab OR nivolumab OR atezolizumab OR durvalumab OR camrelizumab OR toripalimab OR tislelizumab OR sintilimab OR ipilimumab OR avelumab OR cemiplimab OR dostarlimab)

#### 5. Cochrane Library

**Database:** Cochrane Library (CENTRAL and other Cochrane databases)
**Search date:** up to 31 October 2025

**Block 1 – NSCLC with bone metastases (#1):**

( ( "non small cell lung" OR NSCLC OR "non-small cell lung" OR "lung adenocarcinoma" OR "lung squamous cell carcinoma" ) AND ( bone OR skeletal OR spine OR vertebral OR osseous ) AND ( metasta* OR secondar* OR lesion* ) ) OR ( "bone metastas*" OR "skeletal metastas*" OR "spinal metastas*" OR "vertebral metastas*" )

**Block 2 – Radiotherapy (#2):**

radiotherap* OR radiotherapy OR irradiation OR "radiation therapy" OR RT OR SBRT OR SABR OR "stereotactic ablative radiotherapy" OR "stereotactic body radiation therapy" OR IMRT OR "intensity modulated radiation therapy" OR radiosurgery OR "gamma knife" OR "cyberknife" OR "palliative radiation" OR "local therapy"

**Block 3 – Immunotherapy / ICI (#3):**

immunotherap* OR "immune checkpoint" OR ICI OR "checkpoint inhibitor*" OR "checkpoint block*" OR "PD-1" OR "programmed cell death 1" OR "PD L1" OR "programmed death ligand 1" OR "CTLA-4" OR "cytotoxic T-lymphocyte antigen 4"

OR "anti-PD-1" OR "anti-PD-L1" OR "anti-CTLA-4" OR pembrolizumab OR nivolumab OR atezolizumab OR durvalumab OR camrelizumab OR toripalimab OR tislelizumab OR sintilimab OR ipilimumab OR avelumab

**Final combination:**

#1 AND #2 AND #3
