# Supplementary material for: Radiotherapy plus immune checkpoint inhibitors versus immune checkpoint inhibitors alone for non-small cell lung cancer with bone metastases: a systematic review and meta-analysis of comparative cohort studies
Source: Front Immunol. 2026 Feb 19;17:1773998. doi: 10.3389/fimmu.2026.1773998 (PMC12960617; doi:10.3389/fimmu.2026.1773998)
Supplement: Supplementary file 2 [file Table2.docx]

Supplementary Table S1. Radiotherapy and immunotherapy characteristics of included studies

| Study (year) | RT site / target | RT dose–fractionation | RT timing vs ICI | ICI regimen / drug type | Reporting notes |
| --- | --- | --- | --- | --- | --- |
| Asano et al. (2025) [27] | Single bone metastasis treated; most commonly spine; pelvis also frequent | 2–8 Gy per fraction, 1–15 fx (total 8–39 Gy) | 66.7% RT before ICI; remainder after ICI initiation (subgroup) | PD‑1: nivolumab, pembrolizumab; PD‑L1: atezolizumab, durvalumab | Excluded patients receiving RT to lung/brain; excluded bone‑modifying agents |
| Beyon et al. (2025) [28] | Not reported in detail (real‑world database cohort) | Not reported | Mixed: pre‑ICI 55%, during‑ICI 38%, same‑day 7% | PD‑1/PD‑L1 inhibitor monotherapy (e.g., pembrolizumab/nivolumab/atezolizumab) | Largest cohort; heterogeneous RT sequencing and incomplete RT technical details |
| Bozorgmehr et al. (FORCE trial, 2025) [26] | Clinically indicated metastatic lesions other than lung/brain; could include bone (e.g., thoracic spine/chest wall) | 20 Gy in 5 fractions (5×4 Gy), CT‑planned (ICRU 50/62/83) | RT started 72 h after first nivolumab dose; delivered over workdays | Nivolumab 240 mg fixed dose q2 weeks (PD‑1 inhibitor) | Non‑randomized phase II; RT indication-driven allocation |
| Facilissimo et al. (2025) [29] | Symptomatic bone metastases (palliative RT) | 8 Gy×1 (50%) or 20 Gy/5 fx (50%) | During‑ICI only (concomitant RT and ICI) | Anti‑PD‑1 (nivolumab or pembrolizumab) and/or anti‑PD‑L1 (atezolizumab); some with platinum chemo | Mono‑institutional, small RT subgroup (n=10) |
| Qiang et al. (2022) [30] | Bone metastases: spine, ribs, pelvis, sternum, skull, long bones | Commonly 20–30 Gy in 5–10 fx; single‑fraction 8 Gy also used (exact range not fully reported) | During‑ICI only | Pembrolizumab ± platinum‑based chemotherapy (PD‑1 inhibitor) | RT schedule heterogeneous; dose range partly unreported |
| Ratnayake et al. (2019) [31] | Any RT for NSCLC (intracranial or extracranial) | Pre‑ICI: median 30 Gy (8–66); During‑ICI: median 20 Gy (6–50); 1–33 fx | ‘Prior’ >1 week before nivolumab start; ‘During’ within 1 week of starting or while on nivolumab | Nivolumab 3 mg/kg IV q2 weeks (PD‑1 inhibitor) | RT not limited to bone sites; timing windows defined pragmatically |
| RT, radiotherapy; ICI, immune checkpoint inhibitor; BED, biologically effective dose; fx, fractions; PD-1, programmed cell death protein 1; PD-L1, programmed death-ligand 1. | | | | | |
